# A Proposed Model of a Pragmatic Surgical Approach in Women Affected by Uterine Fibroids Undergoing IVF: A “Real Practice” Experience

**DOI:** 10.3390/jcm15010379

**Published:** 2026-01-04

**Authors:** Domenico Antonaci, Francesco Galanti, Roberta Dall’Alba, Eleonora Benedetti, Andrea Rago, Laura Antonaci, Donatella Miriello, Rocco Rago

**Affiliations:** Reproductive Physiopatology and Andrology Unit, Sandro Pertini Hospital, 00157 Rome, Italy; domenico.antonaci@aslroma2.it (D.A.); roberta.dallalba@aslroma2.it (R.D.); eleonora.benedetti1993@gmail.com (E.B.); andrearago2002@gmail.com (A.R.); laura.antonacii@gmail.com (L.A.); donatella.miriello@aslroma2.it (D.M.); rocco.rago@aslroma2.it (R.R.)

**Keywords:** In Vitro Fertilization (IVF), myomas, laparotomic myomectomy, hysteroscopic myomectomy, implantation rate (IR)

## Abstract

**Background/Objectives**: Uterine fibroids are the most common benign neoplasms of the female genital tract, with a prevalence of 20% to 40% among women of reproductive age. Their management in the context of Assisted Reproductive Technologies (ART) represents a major clinical challenge, characterized by controversies, contrasting approaches, and a lack of shared guidelines. Indeed, the detrimental effects of fibroid treatments are not well known and may be influenced by the size, location, and number of fibroids. The impact of hysteroscopic myomectomy in women affected by submucosal myomas (FIGO classification type: G0–G2) is well documented in the current literature; however, the impact of intramural and subserosal myoma removal (FIGO types 3–8), in particular those <4/5 cm in diameter, remains controversial. The aim of the present study is to introduce and share a pragmatic surgical approach to uterine fibroid management prior to In Vitro Fertilization (IVF), to reduce the knowledge gap regarding uterine fibroid treatment. **Methods**: We conducted a retrospective observationally study that included 94 cases of infertile women, who underwent myomectomy at our IVF centre at Sandro Pertini Hospital, Rome, Italy, between 2020 and 2025. These patients met the inclusion criterion of having an idiopathic/tubal factor of infertility and were aged < 40. We evaluated a group of 17 women (group A) who underwent hysteroscopic myomectomy for submucosal fibroids (FIGO types 0–2) and a group of 39 women (group B) who underwent open (laparotomic) myomectomy for intramural/subserosal fibroids (FIGO types 3–8). Group B was compared with a control group of 38 women who were similar in terms of all demographic and clinical parameters and myoma features (group C) and did not want to undergo a myomectomy procedure. All surgical procedures were executed by the same expert surgeon following our proposed model: submucosal fibroids were always removed by operative hysteroscopy, while intramural/subserosal fibroids were removed if there were three or more and if they were at least 1 ≥ 3 cm in size. All enrolled patients subsequently underwent IVF treatment at our centre, which consisted of an antagonist protocol for ovarian stimulation, and all transferred embryos were of good quality according to the recent European Society of Human Reproduction and Embryology (ESHRE) classification. **Results**: In group A, we observed an implantation rate of 41% and a clinical pregnancy rate of 35.2%, and these results are consistent with the current literature. In group B, we obtained statistically significant differences in the implantation (31% vs. 12.9%) and pregnancy rates (28.1% vs. 7.8%) compared to group C (*p* = 0.02 and *p* = 0.03, respectively). In addition, the live birth rate was statistically higher compared to that in group C (*p* < 0.01). Miscarriage and preterm delivery rates were lower in group B, although the differences were not statistically significant. No severe post-surgical complications, such as uterine rupture, were observed during subsequent pregnancies. **Conclusions**: Despite the limited patient sample size, the monocentric experience, and the retrospective design, we emphasize the effectiveness of our proposed surgical model in women affected by myomas. Indeed, the surgical treatment of submucosal, intramural, and subserosal lesions may improve ART and pregnancy outcomes (through a higher implantation rate, pregnancy rate, and live birth rate, as well as a lower miscarriage/preterm rate).

## 1. Introduction

Uterine fibromatosis (or uterine myomas) is a benign condition characterized by the growth of muscle nodules within the uterus and is one of the most common pathologies of the female reproductive tract, with a prevalence among women of childbearing age of around 20–30% and a higher incidence in African American ethnicities [1,2].

The physiopathology of this gynecological disease is not completely understood; indeed, fibroid size may increase or decrease over the years and is influenced by female hormones such as estrogen and progesterone, which are elevated during the reproductive age. Fibroids are frequently asymptomatic and incidentally discovered during ultrasounds or routine gynecological checkups. However, in many cases, they may cause symptoms such as abnormal bleeding, anemia, pelvic pain, and infertility [3].

In recent years, different non-pharmacological and pharmacological approaches have been investigated, such as nutraceutics (e.g., vitamin D and epigallocatenin gallate) or inhibitors of estro-progesterone activity such as ulipristal acetate, but those approaches are usually characterized by frequent relapses and several side effects [4,5,6,7,8].

About the surgical approach, laparoscopic myomectomy is currently considered the gold standard for most fibroid surgeries, including cervical myomas. This technique may be minimally invasive, while open (laparotomic) myomectomy may be reserved for selected complex cases, and it is characterized by longer post-operative recovery times [9,10,11,12].

The rationale for the association between fibroids and infertility is not supported by clear biological evidence and is often explained by bias rather than causality. Despite some clinicians believing that fibroids do not interfere with reproductive outcomes and Assisted Reproductive Technologies (ART), growing evidence suggests that submucosal and intramural fibroids with a cavitary component may significantly compromise implantation and pregnancy rates [13].

Indeed, uterine fibromatosis can negatively affect fertility and the outcome of pregnancies: myomas can distort the uterine cavity, impairing embryo implantation, while intramural–subserosa myomas can increase uterine contractions, causing miscarriage or premature deliveries. Unfortunately, limited attention has been paid to the mechanisms through which fibroids may affect subfertility, usually due to alterations of the uterine cavity contour, leading to mechanical pressure or to the occurrence of abnormal uterine contractility [14,15]. Local inflammation associated with the presence of submucosal fibroids, may negatively impact the endometrial environment, impairing sperm transport and reducing the blood supply, thus impairing the embryo’s invasion mechanism [16,17]. If fibroids are localized near the cervix or the tubal ostia, the anatomical distortion may reduce access of ejaculated sperm to the tubes, whereas large corneal lesions (as well as those characterized by a diameter of less than <4 cm) might impair ovum retrieval by the tubal fimbriae, as well as during ovarian pick-up. Whether women with fibroids > 4 cm would benefit from myomectomy remains to be determined [18].

Indeed, the management of this pathological condition in the context of ART constitutes a significant clinical challenge that is characterized by controversies and contrasting approaches.

Recent studies, such as the meta-analysis by Pritts et al. and the systematic review by Sunkara et al., have demonstrated a reduction in clinical pregnancy and live birth rates in patients with submucosal and intramural fibroids [19,20].

The lack of unanimous consensus among the main national and international scientific societies—such as the European Society of Human Reproduction and Embryology (ESHRE) or the National Institute for Health and Care Excellence (NICE)—as well as standardized protocols, has led to significant variability in clinical practice, creating uncertainty among both professionals and patients. In this context, the implementation of internal guidelines in IVF departments, which can be shared among centres, may help clinicians in the management of these common and complex conditions.

The present observational study aims to evaluate the impact of surgical removal of uterine fibroids on the chances of pregnancies and the outcomes in women undergoing IVF by assessing the effectiveness of our proposed surgical model in improving ART outcomes (e.g., the implantation rate and the live birth rate). In this study we present our surgical guidelines, which are based on careful risk stratification and a differentiated therapeutic approach according to the type, size, and location of fibroids, contributing to the reduction in the existing gap in the current scientific literature regarding this treatment.

## 2. Materials and Methods

We conducted a retrospective observational study involving infertile women affected by uterine fibroids who were treated at the IVF centre of the Physiopathology and Andrology Unit of Sandro Pertini Hospital in Rome, Italy, between 2020 and 2025.

Inclusion criteria were as follows: women aged between 25 and 40 years undergoing IVF at our centre who were affected by asymptomatic uterine myomas and diagnosed with tubal and idiopathic infertility.

Exclusion criteria included the following: endometriosis and adenomyosis, uterine malformations, a male factor (oligo-astenospermia/azoospermia) of infertility, systemic chronic diseases (such as diabetes, hypertension, and thyroid disorders), or previous pelvic/uterine surgery.

We exclusively recruited women who underwent a complete diagnostic work-up at our fertility centre: serum evaluation of FSH and anti-Mullerian (AMH) hormones and hysterosalpingo-contrast sonography (HyCoSy) to assess tubal patency and hysteroscopy (to evaluate a possible distortion of the endometrial cavity). Semen analyses were performed and interpreted based on the 2020 World Health Organization (WHO) recommendations. In addition, all women systematically underwent transvaginal ultrasonography in a dedicated setting, which was exclusively performed by a physician with expertise in transvaginal ultrasonography, to standardize the evaluation of the pelvis. Specifically, all fibroids were recorded in terms of size, dimension, and vascularization according to the Morphological Uterus Sonographic Assessment (MUSA) group’s guidelines [21]. Their location was described according to the revised International Federation of Gynecology and Obstetrics (FIGO) classification [22].

The average dimension of each single fibroid was calculated as the mean of the three perpendicular diameters (in centimetres).

**Figure 1:**
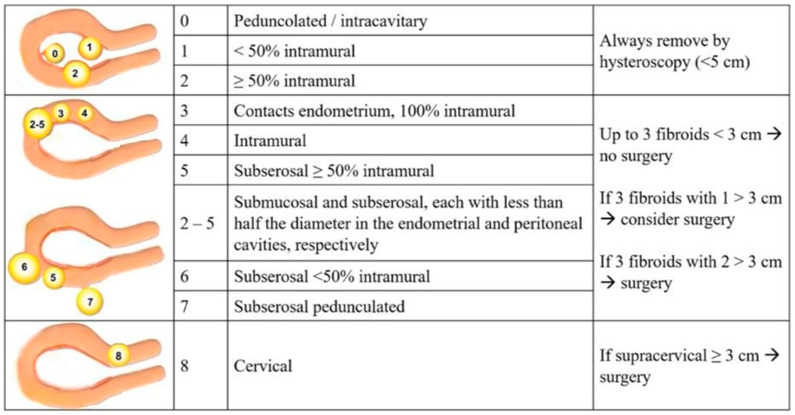
Proposed surgical protocol for uterine fibroid treatment according to D. Antonaci et al. Surgery consisted of a laparotomic approach, but it can also be feasible via laparoscopy, such as in specific cases of cervical myomas.

Our surgical protocol for the uterine fibroid treatment is reported in Figure 1: Myomas of the G0–G2 types under the FIGO classification underwent hysteroscopic myomectomy (group A) if <5 cm. Myomas of the 3–8 types according to FIGO classification, specifically in cases of three intramural/subserosal fibroids with at least one ≥ 3 cm, underwent laparotomic myomectomy (group B).

Specifically, we report the description of the surgical laparotomic technique for intramural and subserosal myomas: a mini-laparotomy incision (usually no more than a 6 cm) with direct visualization of the abdominal cavity, the uterus, and the adnexa; direct palpation of the fibroids; identification of the enucleation plane; use of single hysterotomy for multiple fibroids and utilization of a barbed suture in a layered fashion. The aim is to avoid injury to the fallopian tubes and to other critical uterine structures, while simultaneously repairing the endometrial cavity if damaged. The surgical technique applied was conducted by the same expert surgeon, and all the removed fibroids were subjected to histological examination.

All enrolled women underwent IVF procedures at our fertility centre within 12 months of surgery: at least 1 month after hysteroscopy or 4–6 months after laparotomic myomectomy. Group B was compared to a control group with comparable demographics and hormonal and IVF profiles (group C) who refused the surgical procedure and underwent the IVF stimulation protocol at our centre. Regarding IVF stimulation, all participants were at the first IVF cycle and underwent controlled ovarian stimulation, according to the antagonist stimulation protocol using their own oocytes and sperm. On days 2–3 of their menstrual cycle, patients received recombinant FSH alone, considering age, BMI, FSH, and AMH. Patients were monitored starting from the fifth day of stimulation every two days by measuring estradiol, progesterone, and ultrasound control. When the ultrasound and hormonal parameters were likely to be compatible with oocyte maturity, the trigger was induced by the administration of 10.000 IU of HCG or 0.2 mg/mL triptorelin, and the ovarian pick-up (OPU) procedure was performed 32–36 h after trigger administration. One hour after decumulation, the oocytes deemed suitable by our team of biologists (oocytes in the MII stage) were subjected to the intracytoplasmic sperm injection (ICSI) technique. Normal fertilization (fertilization rate—FR) was identified by the presence of two pronuclei (2PN) at the time of fertilization assessment, which was observed 16 to 19 h after ICSI, using the invertoscope equipped with the Hoffman contrast system and with software for archiving images; all autologous transferred embryos (between days 3–5 of maturation) were of good quality according to ESHRE guidelines and classification [23]. We considered the following outcomes in all three groups: demographics and hormonal parameters, myoma features (localization, number, and size), and IVF data (e.g., number of oocytes and embryos obtained). The implantation rate, clinical pregnancy rate, live birth rate (number of live births divided by number of clinical pregnancies) and pregnancies complications, such as the miscarriage rate, preterm deliveries, and the uterine rupture rate, were considered too. The types of deliveries (spontaneous or caesarean section) were also evaluated.

The present study was conducted following the Ethical Principles of the Helsinki Declaration and the national laws and was approved by the Ethical Committee of our healthcare company, protocol No. 0061226/2020 of 08/04/2020. Regarding the statistical analysis, the present study is a retrospective observational study: Continuous variables were summarized using the mean ± standard deviation (SD) or the median with the interquartile range (IQR), as appropriate. Categorical variables were presented as counts and percentages. Comparisons between two groups for continuous variables were performed using the independent sample *t*-test. Differences in categorical variables between groups were assessed using the chi-square test. All analyses were performed using the SAS software (release 9.4) (Milano, Italy). A *p*-value ≤ 0.05 was considered statistically significant.

## 3. Results

Of the 139 patients affected by uterine myomas referred to our department during the studying period, 45 did not fulfil the inclusion criteria.

The remaining 94 patients were divided in three groups, and the surgical approach was performed according to Figure 1. Group A consisted of 17 patients who underwent hysteroscopic myomectomy, and all the clinical features and obstetrical results are shown in Table 1. The 39 patients affected by myomas of the 3–8 types according to the FIGO classification were subjected to laparotomic myomectomy (group B) and compared with a group of 38 women who were similar in terms of all demographic and clinical features and did not want myomectomy (group C). All demographics and clinical data are represented in Table 2. All myoma characteristics were compared between groups B and C and are reported in Table 3, such as their dimension, number, and localization.

In group A no intra- or post-operative complications occurred. In group B we reported only three cases (9.3%) of wound infections, which were successfully managed. No hemorrhage or damage to adjacent organs (bowel or bladder lesions) occurred. All histological examinations confirmed the diagnosis of leiomyoma and the absence of cellular atypia.

IVF results and pregnancy outcomes compared between groups B and C are reported in Table 4. In group B we reported that eleven (28.1%) patients achieved pregnancies within 12 months of surgery, compared with three (7.8%) patients in group C, and this result is statistically significant. We also underline that 100% of the pregnancies in group B were healthy, while in group C, three patients reported complications such as a miscarriage (one case) and preterm births (two cases). Moreover, the LBR was statistically higher in group B compared to that in group C (*p* < 0.01). No uterine rupture after myomectomy was observed during the study period or throughout pregnancy evolution, and no fibroid recurrence was detected in all treated patients.

All patients in group B underwent a caesarean section (in our obstetrical department, all the previous laparotomic myomectomies are subjected to a caesarean section), while three had spontaneous deliveries (two refused an elective caesarean section, while one had a hasty labour). On the other hand, in group C, two patients underwent a caesarean section: one due to abnormal cardiotography and another due to labour dystocia cause by a voluminous cervical myoma, which increased during the pregnancy’s evolution (from 4–5 cm in diameter to more than 8 cm).

## 4. Discussion

Uterine myomas are a widespread and multifactorial disease that may impact the onset and correct evolution of both spontaneous and ART pregnancies. The biological mechanisms involved are not yet fully understood, although the influence of fibroid size, number, and localization on reproductive outcomes has been demonstrated in the current literature. The impact of submucous fibroids on the distortion of the endometrial cavity and subsequent embryo impairment is well known, and hysteroscopic removal is mandatory in the case of submucous lesions [24,25]. Our results in group A, in which we observed six (35.2%) clinical pregnancies after hysteroscopic removal and the subsequent IVF treatment, are consistent with those of the current literature (Table 1). On the other hand, the negative impact of intramural and subserosal myomas is still not completely known; most studies only evaluate small intramural fibroids (usually considered <3 cm in diameter), probably because women with larger fibroids were excluded and underwent directly laparotomic myomectomy. Hence, the published literature may underestimate the impact of intramural fibroids on the endometrial cavity, particularly the larger ones. Oliveira et al. found a detrimental impact in the presence of relatively larger fibroids. The authors reported lower clinical pregnancy rates after ICSI treatment in women with intramural or subserosal fibroids > 4 cm in diameter compared with women with no fibroids or fibroids ≤ 4 cm [26]. Moreover, they found no difference in pregnancy rates between the control group and women with fibroids ≤ 4 cm, although women with fibroids ≥ 7 cm were excluded. Another retrospective study evaluating non-distorting fibroids found that delivery rates were lower in the presence of fibroids > 2.85 cm, whilst there was no detrimental impact on the endometrial cavity in the presence of smaller fibroids [27]. A more recent retrospective matched cohort study showed that fibroids ≥ 3 cm had a deleterious effect on live birth rates, whereas this effect was not observed in the presence of fibroids < 3 cm [28]. In light of these studies, we designed our proposed surgical model by tailoring a cut-off diameter for surgical removal of 3 cm (Figure 1). Specifically, we tried to avoid surgery in the presence of fibroids < 3 cm when the uterine cavity was regular, proposing surgery only to women with three intramural/subserosal fibroids ≥ 3 cm. In the case of fibroids with a diameter of 4 cm or more, we recommended surgery. Additionally, to avoid difficulties with ovarian accessibility during ovum pick-up and due to intramural/subserosal lateral fibroids, we always prefer to perform myomectomy before IVF.

This surgical criterion is based on volumetric and functional considerations for uterine physiology and fertility: the presence of three fibroids, two of which are ≥3 cm in diameter, results in a total volume of approximately 28–30 cm^3^. Considering an ideal uterus with an average volume of approximately 80–90 cm^3^, the presence of these fibroids represents an increase in the total volume of the uterus of at least 30%, thus significantly impacting its overall size. A single fibroid with a volume of 30 cm^3^ exerts a localized and concentrated pressure on the surrounding myometrium and uterine structures. This can lead to focal deformation, vascular compression, and disruption of peristaltic contractility, impairing embryo implantation and endometrial receptivity. In contrast, multiple fibroids distributing the same total volume exert their mechanical load across multiple sites. Although the pressure at each site may be lower, the diffuse mechanical stress can lead to global alterations in uterine tone, disorganization of myometrial contractions, focal vascular disturbances, and altered neurological signals essential for successful conception. Additionally, from a geometrical and biomechanical standpoint, three fibroids with a total volume of 30 cm^3^ have a significantly greater total surface area, approximately 44% more, compared to a single fibroid with the same volume. This increased surface contact with the uterine tissue amplifies the extent of local myometrial interaction and mechanical disruption, potentially worsening the functional impact on uterine receptivity and coordinated contractility. All these motivations guide the drafting and application of our surgical guideline.

Our results align with another study by Yan et al., who suggested that FIGO type 3 fibroids exert a negative impact on the rates of implantation, clinical pregnancy, and live birth in patients undergoing IVF-ICSI, but do not significantly increase the clinical miscarriage rate; the deleterious impact of type 3 fibroids was remarkable in women with fibroids > 2.0 cm in diameter [29]. Our results are also consistent with those of Sunkara et al., where the analysis of 19 studies on the impact of non-cavity distorting intramural fibroids on IVF outcomes found significant reductions in live birth rates (OR: 0.79 and 95% CI: 0.70–0.88) and clinical pregnancy rates (OR: 0.85 and 95% CI: 0.77–0.94) in women with fibroids compared with women without fibroids, although the implantation and miscarriage rates were not statistically different. All the studies included in this review article report data from women with fibroids of 0.4–8 cm in diameter, with the majority being less than 5 cm of diameter [15]. Our results are also in accord with those of Bulletti and Sarıdoğan E, in which the authors found a negative effect of intramural fibroids on IVF pregnancies, despite not impacting the endometrial cavity [30,31]. Moreover, another recent work by Favilli et al. including 1020 women, of which 324 were affected by FIGO type 3, highlighted that the number and size of fibroids correlated with a worsening of IVF outcomes, reporting significantly lower live birth, clinical pregnancy, and implantation rates in women with untreated myomas compared with controls [32]. Another recent systematic review and meta-analysis stated that non-cavity-distorting intramural fibroids ≤ 6 cm in size, negatively impacted IVF outcomes [33]. A recent study focusing on IVF and embryo transfer, highlighted comparable results in terms of clinical pregnancy rates between treated and untreated fibroid groups [34]. All those results are consistent with ours, in which we reported a higher pregnancy rate in the treated group (Table 4). In contrast with our data, two retrospective studies suggest that small fibroids that do not encroach the endometrial cavity, do not markedly affect female fertility; however, in those studies, the authors did not evaluate myomas larger than 6 cm in diameter, while in our work, we also evaluated bigger ones (Table 3). Moreover, those two studies included women older than 40 years, where a reduced ovarian reserve could represent a bias [35,36]. Despite these facts, both works conclude that the surgical approach may still be considered, but only in selected cases. A systematic review by the American Society for Reproductive Medicine (ASRM) stated that there is insufficient evidence to conclude that the presence of myomas reduces the likelihood of achieving pregnancy, but there is evidence that myomectomy (open or laparoscopic) for cavity-distorting myomas (intramural or intramural with a submucosal component) improves pregnancy rates and reduces the risk of early pregnancy loss [37]. The ASMR conclude that in women affected by asymptomatic cavity-distorting myomas, myomectomy may be considered to optimize pregnancy outcomes.

Regarding miscarriage and preterm delivery rates, our data showed no statistically significant differences between groups B and C (only two cases in group C); we believe that this minor difference is due to the very low number of patients recruited. Moreover, in group C we reported two caesarean sections, one of them due to a bulky cervical myoma that doubled in volume during the pregnancy’s evolution. As matter of fact, this kind of myoma may be very dangerous for both woman’s and unborn baby’s health.

**Figure 2:**
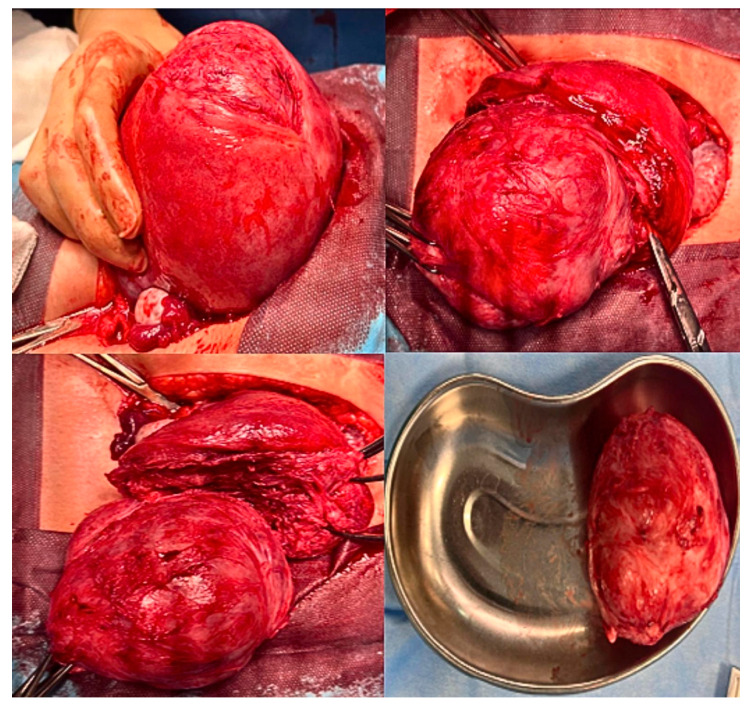
Single myomectomy: The pictures represent a case of a single huge intramural myoma removed by a mini-laparotomy incision.

**Figure 3:**
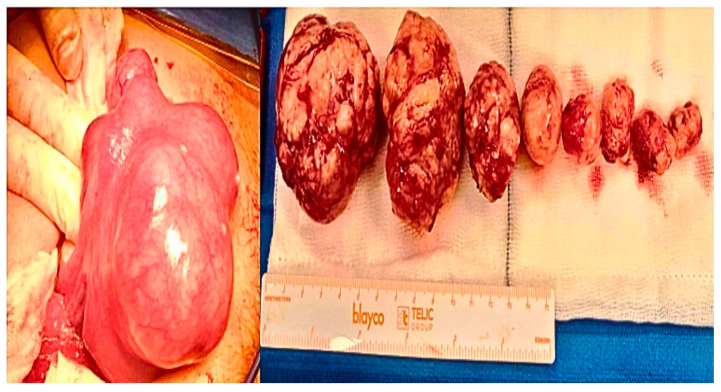
Multiple myomectomy: The pictures illustrate a severe deformed uterus caused by the presence of numerous intramural and subserosal myomas, which were completely removed.

Regarding the strength of our study, we choose to follow the FIGO classification system in our guidelines in order to standardized the description of the fibroids’ relationships with the endometrial cavity, the myometrium, and the uterine serosa [38]. Regarding the choice to perform hysteroscopic myomectomy in the case of G0–G1 or G2 myomas < 5 cm, we followed the Italian guidelines regarding fibroids treatment, which was published in 2017 by the Italian University Gynaecologist Association (AGUI) [39]. We underline that the surgical technique was always performed by the same expert gynecological surgeon (Dr. Antonaci), who preferred to perform a mini-laparotomic incision in cases of myomas from FIGO 3 onwards, due to more confidence and expertise in open surgery. This approach is characterized by a mini-Pfannenstiel incision, a complete enucleation of single or multiple fibroids, and a continuous multilayer uterine wall suture. This reduces the surgical knots, as well as the number of post-operative adhesions (Figure 2 and Figure 3); the present suturing technique avoids the endometrial cavity and therefore its exposure to foreign bodies such as sutures, decreases intrauterine adhesion formation while increasing the healing of uterine scars, and reduces the risk of uterine rupture [13,40]. We also chose to always perform laparotomic myomectomy, in order to respect the Food and Drug Administration (FDA)’s 2014 statement, which warns against the use of laparoscopic power morcellators [41]. Our surgical algorithm would also certainly be feasible for laparoscopic surgery, specifically in the case of cervical myomas or intramural/sub-serosal ones. We are aware that this technique is the present and future of gynecological surgery, as it is robotic surgery. Indeed, nowadays, laparoscopy is the most used minimally invasive approach for the treatment of several gynecological pathologies, characterized by a low complication rate, and a rapid post-operative recovery of the patient.

However, women were advised to avoid unprotected intercourses and initiate IVF no earlier than 4–6 months after abdominal myomectomy to avoid complications during pregnancy such as uterine rupture. Thanks to this precaution, we did not report uterine ruptures in the treated group. In the hysteroscopic myomectomy group, the IVF technique was performed prior to one month after the treatment, since the integrity of the myometrial structure is not affected in this case. It may be argued that the delay for the beginning of the ART treatment in the case of laparotomic myomectomy may potentially be an issue for older women, particularly for those with reduced ovarian reserve. This delay might be overcome by performing IVF before myomectomy and freezing the embryos for transfer after the recovery period. Despite this fact, we choose to exclude the freeze-all protocol to focus our result only on the surgical technique and on the validity of our protocol while avoiding the detriment of ovarian reserve quality and AMH reduction, which could negatively impact pregnancy outcomes.

Despite the limitations of the present study (small sample of patients involved, scarce number of pregnancies, and monocentric experience), our results emphasize that surgery for the removal of uterine fibroids is an effective option for patients desiring pregnancy. We are also aware that pregnancies obtained by ART have increased potential obstetrical risks, which could be caused by fetus–placenta unit development, which is particularly affected by placentation and several other different etiopathogenetic factors [42]. Although these facts, we trust that our experience during this studying period emphasizes that uterine myoma surgery can lead to satisfactory and positive pregnancy outcomes.

We are aware that this is a very preliminary experience, and not the only guideline to take into consideration in terms of surgical approaches, but it represents a pragmatic starting model to then expand the knowledge of for this widespread condition in women undergoing IVF. Further prospective studies are needed to encourage the investigation of these findings and optimize treatment strategies for women with uterine fibromatosis, in order to standardize this practical surgical model in other national ART centres.

## 5. Conclusions

This study presents the effectiveness of our surgical guidelines, which were based on careful risk stratification and a differentiated therapeutic approach according to the type, size, and location of fibroids in women undergoing ART. Our aim is to reduce the existing gap on this common female condition in the current scientific literature, by providing a pragmatic approach to the management of uterine fibroids prior to ICSI techniques. We aim to share these guidelines with other experts in the surgical and infertility fields and, more importantly, to engage with IVF centres across the nation to conduct multicentre “real practice” studies and establish clear and standardized protocols. The goal is to standardize and improve the approach to uterine myomas in infertile patients to maximize IVF success rates and the safety of achieved pregnancies.

## Figures and Tables

**Table 1:** Demographic and clinical features of 17 women affected by submucosal myomas of the G0–G2 types according to the FIGO classification (group A) who underwent hysteroscopic myomectomy and subsequently IVF treatment. All data are represented as the mean and standard deviation (SD) or number (n°) and percentage (%); a *p*-value ≤ 0.05 is statistically significative; ns: not statically significative.

*Clinical Parameters*	*Mean (SD)*
Age (years)	36.53 ± 3.34
BMI (kg/m^2^)	25.18 ± 2.32
Duration of infertility (years)	3.2 ± 1.2
Basal FSH (mIU/mL)	6.19 ± 2.32
AMH (ng/mL)	1.26 ± 0.77
Days of hormonal stimulation	13.12 ± 2.06
Total dose of gonadotropin (UI)	2077.35 ± 999.15
MII Oocytes retrieved	4.29 ± 1.86
Embryos obtained	1.76 ± 1.15
***Clinical parameters and pregnancy outcomes***	***n° (%)***
Tubal factor of infertility	10 (58.8)
Idiopathic factor of infertility	7 (41.2)
Implantation rate	7 (41%)
Clinical pregnancy (CPR)	6 (35.2%)
Live birth rate (LBR)	6 (100%)
Miscarriage	1 (5.8%)
Preterm delivery	0 (0%)
Caesarean section (of total CPR)	3 (50%)

**Table 2:** Demographic and clinical features as well as IVF results of women affected by myomas of the 3–8 types according to the FIGO classification, between group B (myomectomy) vs. group C (no myomectomy). All data are represented as the mean and standard deviation (SD) or number (n°) and percentage (%); a *p*-value ≤ 0.05 is statistically significative; ns: not statically significative.

*Demographics and Clinical Parameters*	*Group B (n° 39)*	*Group C (n° 38)*	*p*-*Value*
Age (years) Mean (SD)	38.88 ± 3.38	38.84 ± 4.03	Ns
BMI (kg/m^2^) Mean (SD)	24.71 ± 5.23	23.2 ± 3.75	0.19
Duration of infertility (years) Mean (SD)	2.1 ± 1.2	2.2 ± 1.1	Ns
Basal FSH (mIU/mL) Mean (SD)	7.15 ± 2.20	6.55 ± 2.01	0.27
AMH (ng/mL) Mean (SD)	1.20 ± 0.79	1.10 ± 0.78	Ns
Tubal factor of infertility n° (%)	15 (38.5)	16 (42)	Ns
Idiopathic factor of infertility n° (%)	24 (61.5)	22 (58)	Ns
MII Oocytes retrieved (n°) Mean (SD)	3.66 ± 1.81	3.60 ± 1.85	Ns
Embryos obtained (n°) Mean (SD)	1.16 ± 0.99	1.13 ± 1.02	Ns

**Table 3:** Myomas features of women affected by myomas type 3–8 FIGO classification, confronted between group B (myomectomy) vs. group C (no myomectomy). All data are represented as number (n°); *p*-value ≤ 0.05 is statistically significative; ns: not statically significative.

*Myomas Localization (According to FIGO 2011 Classification)*	*Group B (n° 39)*	*Group C (n° 38)*	*p-Value*
Type 3	10	11	Ns
Type 4	13	12	Ns
Type 5	18	18	Ns
Type 6	18	7	Ns
Type 7	4	5	Ns
Type 8	5	3	Ns
***Dimensions (cm)***			
Small (<3)	10	5	Ns
Medium (3–5)	49	39	Ns
Large (>5)	6	3	Ns
Min. dimension	2	2	Ns
1st Quartile	3	3	Ns
Median	4	4	Ns
Mean	4.1	3.7	Ns
3rd Quartile	5	4.5	Ns
Max. dimension	12	8	Ns
***Typologies of myomas***			
Single	23	28	Ns
Multiple	23	18	Ns

**Table 4:** Pregnancy outcomes after IVF treatment in women affected by myomas of the 3–8 types according to the FIGO classification, compared between group B (laparotomic myomectomy) vs. group C (no myomectomy). All data are represented as the number (n°) and percentage (%); a *p*-value ≤ 0.05 is statistically significative; ns: not statically significative.

*Pregnancy Outcomes*	*Group B (n° 39)*	*Group C (n° 38)*	*p-Value*
Pregnancy positive test n° (%)	12 (31)	5 (13)	0.03
Clinical pregnancy n° (%)	11 (28.1)	3 (7.8)	0.02
Miscarriage (<23 weeks) n° (%)	1 (3.9)	1 (3.9)	Ns
Preterm deliveries n° (%)	0 (0)	2 (5.2)	Ns
Spontaneous deliveries n° (%)	3 (7.6)	1 (3.8)	Ns
Caesarean section n° (%)	8 (20.5)	1 (3.8)	<0.01
Live Birth n° (%)	11 (100)	1 (25)	<0.01
Uterine rupture n° (%)	0 (0)	0 (0)	Ns

## Data Availability

All data are unavailable due to privacy policies.

## Abbreviations

The following abbreviations are used in this manuscript:

IVF	In Vitro Fertilization
ART	Assisted Reproductive Technologies
ICSI	Intracytoplasmic Sperm Injection
WHO	World Health Organization
ESHRE	European Society of Human Reproduction and Embryology
NICE	National Institute for Health and Care Excellence
ASRM	American Society Reproductive Medicine
FIGO	International Federation of Gynecology and Obstetrics
AGUI	Italian University Gynaecologist Association
